# Supplementary material for: End Sequence Analysis Toolkit (ESAT) expands the extractable information from single-cell RNA-seq data
Source: Genome Res. 2016 Oct;26(10):1397–410. doi: 10.1101/gr.207902.116 (PMC5052061; doi:10.1101/gr.207902.116)
Supplement: Supplemental Material [file supp_gr.207902.116_Supp_Materials.tar.gz › ESAT-esat_v0.1/doc/html/classbroad_1_1pda_1_1differential_expression_1_1_differential_expression.html]

ESAT: broad.pda.differentialExpression.DifferentialExpression Class Reference


ESAT


- Main Page
- Related Pages
- Packages
- Classes
- Files

- Class List
- Class Index
- Class Hierarchy
- Class Members

All Classes Namespaces Files Functions Variables Enumerator Pages

- broad
- pda
- differentialExpression
- DifferentialExpression

Public Member Functions |
List of all members

broad.pda.differentialExpression.DifferentialExpression Class Reference

Collaboration diagram for broad.pda.differentialExpression.DifferentialExpression:

[legend]

|  |  |
| --- | --- |
| Public Member Functions | |
|  | DifferentialExpression (MatrixWithHeaders data, Collection< String > group1, Collection< String > group2, boolean useFold, boolean paired, int perm, double[] fudgeFactors, MatrixWithHeaders[] permutationMatrix, Map< String, FDRDistribution > fdrDists) |
|  | |
|  | DifferentialExpression (MatrixWithHeaders data, Map< String, Collection< String >> groups, boolean useFold, boolean paired, int perm, double[] fudgeFactors) |
|  | |
|  | DifferentialExpression (MatrixWithHeaders data, Collection< String > group1, Collection< String > group2, boolean useFold, boolean paired, int perm, double[] fudgeFactors, MatrixWithHeaders[] permutationMatrix) |
|  | |
|  | DifferentialExpression (MatrixWithHeaders data, Collection< String > group1, Collection< String > group2, boolean useFold, boolean paired, int perm) |
|  | |
|  | DifferentialExpression (MatrixWithHeaders data, Collection< String > group1, Collection< String > group2, boolean useFold, boolean paired) |
|  | |
|  | DifferentialExpression (MatrixWithHeaders data, Collection< String > group1, Collection< String > group2) |
|  | |
|  | DifferentialExpression (MatrixWithHeaders data, Collection< String > group1, Collection< String > group2, int perm) |
|  | |
| Map< String, FDRDistribution > | getFDRDistribution () |
|  | |
| MatrixWithHeaders | getTestStatisticMatrix () |
|  | |
| MatrixWithHeaders | getFDRMatrix () |
|  | |
| MatrixWithHeaders | getAbsFDRMatrix () |
|  | |
| MatrixWithHeaders[] | getPermutationMatrix () |
|  | |
| MatrixWithHeaders | getFoldMatrix () |
|  | |
| MatrixWithHeaders | getFWERMatrix () |
|  | |
| MatrixWithHeaders | getNominalPValueMatrix () |
|  | |
| MatrixWithHeaders | getGenesPassingFDR (double alpha) |
|  | |

## Constructor & Destructor Documentation

|  |  |  |  |
| --- | --- | --- | --- |
| broad.pda.differentialExpression.DifferentialExpression.DifferentialExpression | ( | MatrixWithHeaders | *data*, |
|  |  | Collection< String > | *group1*, |
|  |  | Collection< String > | *group2*, |
|  |  | boolean | *useFold*, |
|  |  | boolean | *paired*, |
|  |  | int | *perm*, |
|  |  | double[] | *fudgeFactors*, |
|  |  | MatrixWithHeaders[] | *permutationMatrix*, |
|  |  | Map< String, FDRDistribution > | *fdrDists* |
|  | ) |  |  |

|  |  |  |  |
| --- | --- | --- | --- |
| broad.pda.differentialExpression.DifferentialExpression.DifferentialExpression | ( | MatrixWithHeaders | *data*, |
|  |  | Map< String, Collection< String >> | *groups*, |
|  |  | boolean | *useFold*, |
|  |  | boolean | *paired*, |
|  |  | int | *perm*, |
|  |  | double[] | *fudgeFactors* |
|  | ) |  |  |

|  |  |  |  |
| --- | --- | --- | --- |
| broad.pda.differentialExpression.DifferentialExpression.DifferentialExpression | ( | MatrixWithHeaders | *data*, |
|  |  | Collection< String > | *group1*, |
|  |  | Collection< String > | *group2*, |
|  |  | boolean | *useFold*, |
|  |  | boolean | *paired*, |
|  |  | int | *perm*, |
|  |  | double[] | *fudgeFactors*, |
|  |  | MatrixWithHeaders[] | *permutationMatrix* |
|  | ) |  |  |

|  |  |  |  |
| --- | --- | --- | --- |
| broad.pda.differentialExpression.DifferentialExpression.DifferentialExpression | ( | MatrixWithHeaders | *data*, |
|  |  | Collection< String > | *group1*, |
|  |  | Collection< String > | *group2*, |
|  |  | boolean | *useFold*, |
|  |  | boolean | *paired*, |
|  |  | int | *perm* |
|  | ) |  |  |

|  |  |  |  |
| --- | --- | --- | --- |
| broad.pda.differentialExpression.DifferentialExpression.DifferentialExpression | ( | MatrixWithHeaders | *data*, |
|  |  | Collection< String > | *group1*, |
|  |  | Collection< String > | *group2*, |
|  |  | boolean | *useFold*, |
|  |  | boolean | *paired* |
|  | ) |  |  |

|  |  |  |  |
| --- | --- | --- | --- |
| broad.pda.differentialExpression.DifferentialExpression.DifferentialExpression | ( | MatrixWithHeaders | *data*, |
|  |  | Collection< String > | *group1*, |
|  |  | Collection< String > | *group2* |
|  | ) |  |  |

|  |  |  |  |
| --- | --- | --- | --- |
| broad.pda.differentialExpression.DifferentialExpression.DifferentialExpression | ( | MatrixWithHeaders | *data*, |
|  |  | Collection< String > | *group1*, |
|  |  | Collection< String > | *group2*, |
|  |  | int | *perm* |
|  | ) |  |  |

## Member Function Documentation

|  |  |  |  |  |
| --- | --- | --- | --- | --- |
| MatrixWithHeaders broad.pda.differentialExpression.DifferentialExpression.getAbsFDRMatrix | ( |  | ) |  |

|  |  |  |  |  |
| --- | --- | --- | --- | --- |
| Map<String, FDRDistribution> broad.pda.differentialExpression.DifferentialExpression.getFDRDistribution | ( |  | ) |  |

|  |  |  |  |  |
| --- | --- | --- | --- | --- |
| MatrixWithHeaders broad.pda.differentialExpression.DifferentialExpression.getFDRMatrix | ( |  | ) |  |

|  |  |  |  |  |
| --- | --- | --- | --- | --- |
| MatrixWithHeaders broad.pda.differentialExpression.DifferentialExpression.getFoldMatrix | ( |  | ) |  |

|  |  |  |  |  |
| --- | --- | --- | --- | --- |
| MatrixWithHeaders broad.pda.differentialExpression.DifferentialExpression.getFWERMatrix | ( |  | ) |  |

|  |  |  |  |  |  |
| --- | --- | --- | --- | --- | --- |
| MatrixWithHeaders broad.pda.differentialExpression.DifferentialExpression.getGenesPassingFDR | ( | double | *alpha* | ) |  |

Here is the call graph for this function:

|  |  |  |  |  |
| --- | --- | --- | --- | --- |
| MatrixWithHeaders broad.pda.differentialExpression.DifferentialExpression.getNominalPValueMatrix | ( |  | ) |  |

|  |  |  |  |  |
| --- | --- | --- | --- | --- |
| MatrixWithHeaders [] broad.pda.differentialExpression.DifferentialExpression.getPermutationMatrix | ( |  | ) |  |

|  |  |  |  |  |
| --- | --- | --- | --- | --- |
| MatrixWithHeaders broad.pda.differentialExpression.DifferentialExpression.getTestStatisticMatrix | ( |  | ) |  |

---

The documentation for this class was generated from the following file:

- java/broad/pda/differentialExpression/DifferentialExpression.java


---

Generated on Fri Aug 15 2014 15:10:59 for ESAT by  

 1.8.7
